# Sleep and Epilepsy Link by Plasticity

**DOI:** 10.3389/fneur.2020.00911

**Published:** 2020-08-28

**Authors:** Péter Halász, Anna Szűcs

**Affiliations:** ^1^Szentágothai János School of Ph.D Studies, Clinical Neurosciences, Semmelweis University, Budapest, Hungary; ^2^Institute of Behavioral Sciences, Semmelweis University, Budapest, Hungary

**Keywords:** sleep-related epilepsy, NREM sleep plasticity, system epilepsy, epileptic network, epileptogenesis, epileptic learning

## Abstract

We aimed to explore the link between NREM sleep and epilepsy. Based on human and experimental data we propose that a sleep-related epileptic transformation of normal neurological networks underlies epileptogenesis. Major childhood epilepsies as medial temporal lobe epilepsy (MTLE), absence epilepsy (AE) and human perisylvian network (PN) epilepsies - made us good models to study. These conditions come from an epileptic transformation of the affected functional systems. This approach allows a system-based taxonomy instead of the outworn generalized-focal classification. MTLE links to the memory-system, where epileptic transformation results in a switch of normal sharp wave-ripples to epileptic spikes and pathological high frequency oscillations, compromising sleep-related memory consolidation. Absence epilepsy (AE) and juvenile myoclonic epilepsy (JME) belong to the corticothalamic system. The burst-firing mode of NREM sleep normally producing sleep-spindles turns to an epileptic working mode ejecting bilateral synchronous spike-waves. There seems to be a progressive transition from AE to JME. Shared absences and similar bilateral synchronous discharges show the belonging of the two conditions, while the continuous age windows - AE affecting schoolchildren, JME the adolescents - and the increased excitability in JME compared to AE supports the notion of progression. In perisylvian network epilepsies - idiopathic focal childhood epilepsies and electrical status epilepticus in sleep including Landau-Kleffner syndrome - centrotemporal spikes turn epileptic, with the potential to cause cognitive impairment. Postinjury epilepsies modeled by the isolated cortex model highlight the shared way of epileptogenesis suggesting the derailment of NREM sleep-related homeostatic plasticity as a common step. NREM sleep provides templates for plasticity derailing to epileptic variants under proper conditions. This sleep-origin explains epileptiform discharges' link and similarity with NREM sleep slow oscillations, spindles and ripples. Normal synaptic plasticity erroneously overgrowing homeostatic processes may derail toward an epileptic working-mode manifesting the involved system's features. The impact of NREM sleep is unclear in epileptogenesis occurring in adolescence and adulthood, when plasticity is lower. The epileptic process interferes with homeostatic synaptic plasticity and may cause cognitive impairment. Its type and degree depends on the affected network's function. We hypothesize a vicious circle between sleep end epilepsy. The epileptic derailment of normal plasticity interferes with sleep cognitive functions. Sleep and epilepsy interconnect by the pathology of plasticity.

## Introduction

The strong relationship of sleep and epilepsy has attracted scholars for a long time. In an excellent atlas, the Gibbses (1) described the NREM sleep enhancement of interictal epileptic discharges (IEDs). Several authors (2–4) summarized the related data, mainly on the distribution of spikes and seizures across sleep stages in different epilepsies. The growing knowledge about sleep physiology in the end of the last century increased the interest in sleep grapho-elements and their role in epilepsies. It has turned out that NREM sleep participates in the organization of several brain functions like synaptic homeostasis (5, 6), memory consolidation (7), vigilance regulation (8), and probably also in regeneration processes in general (9). Experimental sleep studies and human experiences with intracranial electrodes during presurgical evaluation provided many new data on the sleep-epilepsy relationship. The view about epilepsy has changed in the last 20–30 years in many respects. Maintaining the “generalized”/“focal” break-up of epilepsies has become increasingly difficult. “Generalized epilepsies” were not enough generalized and “focal epilepsies” not enough focal (10). The concept of “system epilepsy” offers a new frame for taxonomy.

Growing evidence support that epilepsies transform functional brain systems (11–14).

While epilepsy is a heterogeneous condition, brain lesions and genetic abnormalities trigger a common physio-pathomechanism, presumably very close to normal functioning. It leads to various epileptic syndromes.

The development of epilepsies shares several fundamental features. (1) The normal functioning of a brain system turns to an epileptic working mode. (2) The epileptic transformation favors developmental periods. (3) Epilepsy typically affects those brain structures involved in sleep plastic functions e.g., the corticothalamic and the fronto-hippocampal system. (4) A second disease character: there is a “first hit” (early precipitator injury) and a hidden and long-lasting “ripening” period with synaptic (epileptic) reorganization. (5) The ictal symptoms typically magnify (or rarely paralyze) some functions of the affected system.

Several authors have proposed brain plasticity or its abnormality, as a common pathogenic agent. Plasticity is one the most important mode of operation within the central nervous system defined as an “activity-dependent alteration in the strength of connection among neurons, through which information is stored” (15).

Goddard and Douglas (16) was the first supposing that the plastic process of memory trace (engram)-formation and epileptogenesis are similar (17). The repetitive stimulation of a neuron may generate long-term potentiation (LTP) in a synaptically connected second one, strengthening its function [(18), Nobel price]. LTP has become the elementary model of plasticity. Similarly, in kindling, daily electrical stimulation results in the development of an epileptic process leading to spontaneous seizures (19). Chemically or physically induced experimental epileptic foci that bombard distant regions with spikes, may establish secondary interictal spike foci becoming independent later. Such secondary cortical spots “learn to be epileptic” (20) due to a plastic and potentially progressive process induced by recurrent interictal epileptiform discharges (IEDs) (21).

The isolated cortex and hippocampus “operate extremely close to the transition point between a quiescent state and an abnormally active epileptic state” (17, 22).

In this paper, we trace epileptogenesis in four sleep-related major epilepsies hoping to explore the inter-relationship between epilepsy and NREM sleep.

## The Epileptic Transformation of the Hippocampo-Frontal Declarative Memory Network in Medial Temporal Lobe Epilepsy (MTLE)

### Clinical Course and Animal Models

**Figure 1:**
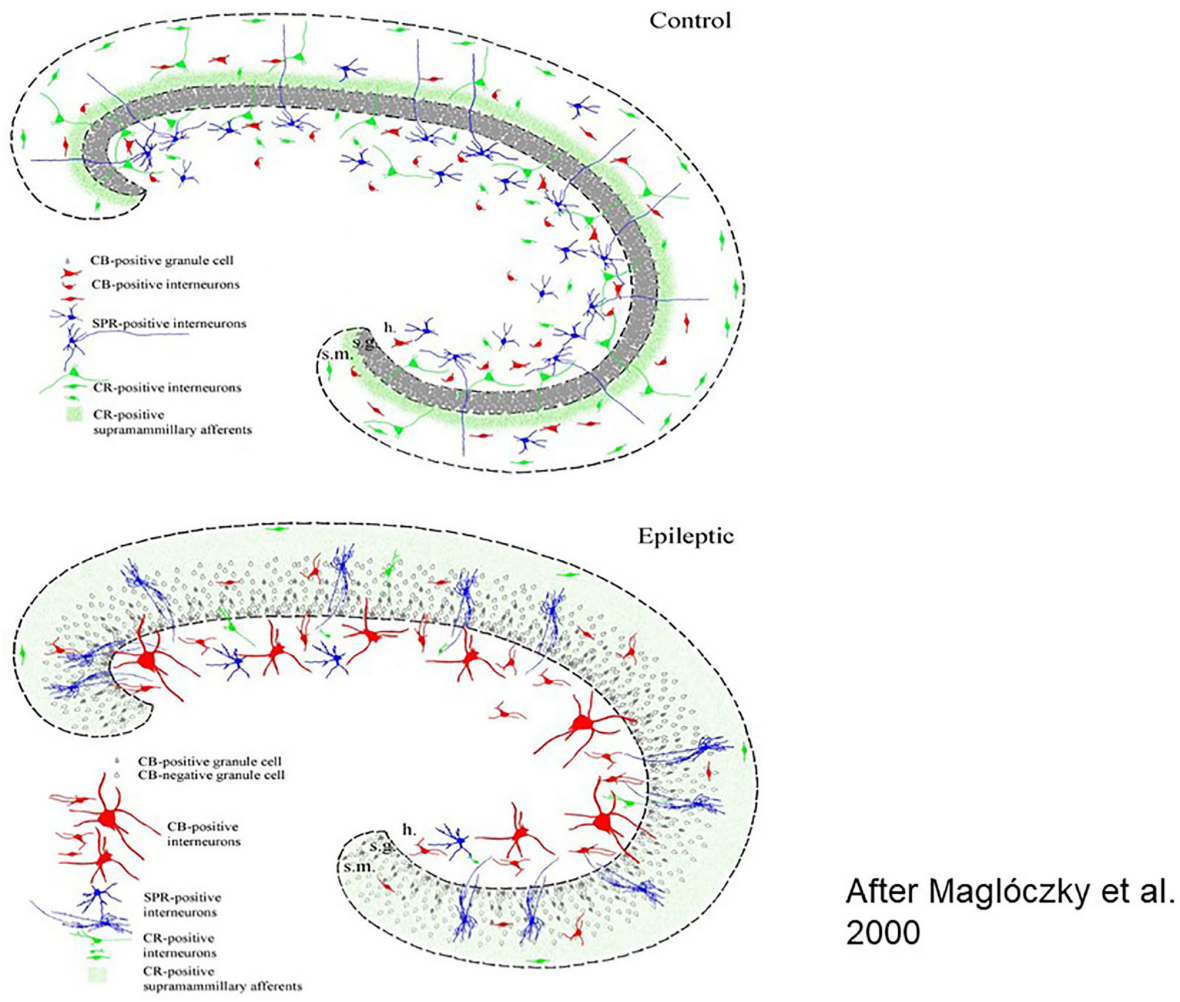
Histologic workup of resected human brain samples (schematic drawing). Reorganization of the hippocampal network after years of epileptic transformation Left: cellular changes, right: interneuronal changes [After (23)].

Medial temporal lobe epilepsy is the most prevalent human epilepsy. There is an early precipitating factor initiating a long epileptogenic process. After a hidden development during childhood, it manifests clinically around puberty becoming frequently resistant to treatment. The common scheme is an early damage in the epilepsy-prone anterior limbic structures (complex febrile seizures, status epilepticus) affecting mainly the CA1 (cornu ammonis 1) and CA3 layers of the hippocampus. The appearance of variable severity hippocampal sclerosis (HS) with synaptic reorganization (Figure 1) and mossy fiber sprouting (24) follows. This years-lasting and hidden progress leads to recurrent temporal seizures and cognitive impairment. Animal experiments and techniques [electrical stimulation, (25); kindling or chemical interventions e.g., Kainic acid: (26, 27); Pilocarpin: (28)] as well investigations of resected human specimens were used for scrutinizing this process.

Several factors such as neuropeptides, neuromodulators and growth factors take part and lead to a severe epileptogenic reorganization of the interneuronal synaptic network structure in the anterior part of the limbic structures and the hippocampus, underlying the variable symptomatology of MTLE.

In addition to primary hippocampal lesions, secondary hippocampal damages may be caused by dysgenetic and other tumors lying in the vicinity or in remote structures connecting with the hippocampi. Posterior temporo-parietal lesions (e.g., peritrigonal nodular dysgenetic), tubers of tuberous sclerosis and lesions in the frame of Sturge-Weber syndrome were described (29).

Since the elaboration of the case of H. M. with bilateral hippocampectomy (30), the essential role of the hippocampi in human declarative memory has become obvious. Neuropsychological testing of surgical MTLE patients and the progress of neuroimaging have contributed to the understanding of the memory process, still limited by the narrow neuropsychology capacity and the lack of follow-up studies. In MTLE, several ictal features reflect the involvement of memory (e.g., déjà-vu and jamais vu experiences, pure amnestic fits etc.) suggesting that acute transient memory losses, rather than genuine disturbances of consciousness may underlie patients' ictal loss of contact in partial seizures. Hippocampal sclerosis appeared to fully account for the side-specific memory disturbance in MTLE, however, the transformation of sharp-wave ripples (SPW-Rs) to interictal epileptiform discharges (IEDs) obstructing memory consolidation, has made an additional factor (31).

### Epileptic Transformation in the Hippocampo-Frontal Memory System

Recognizing the functional damage of the memory system took a long time. The results of memory and sleep research allowed decoding those circuits and working modes underlying memory processing in NREM sleep and the close link with epilepsies.

### The Sharp Wave-Ripple (SPW-R) in NREM Sleep Is a Key Player of the Memory System's Epileptic Transformation

**Figure 2:**
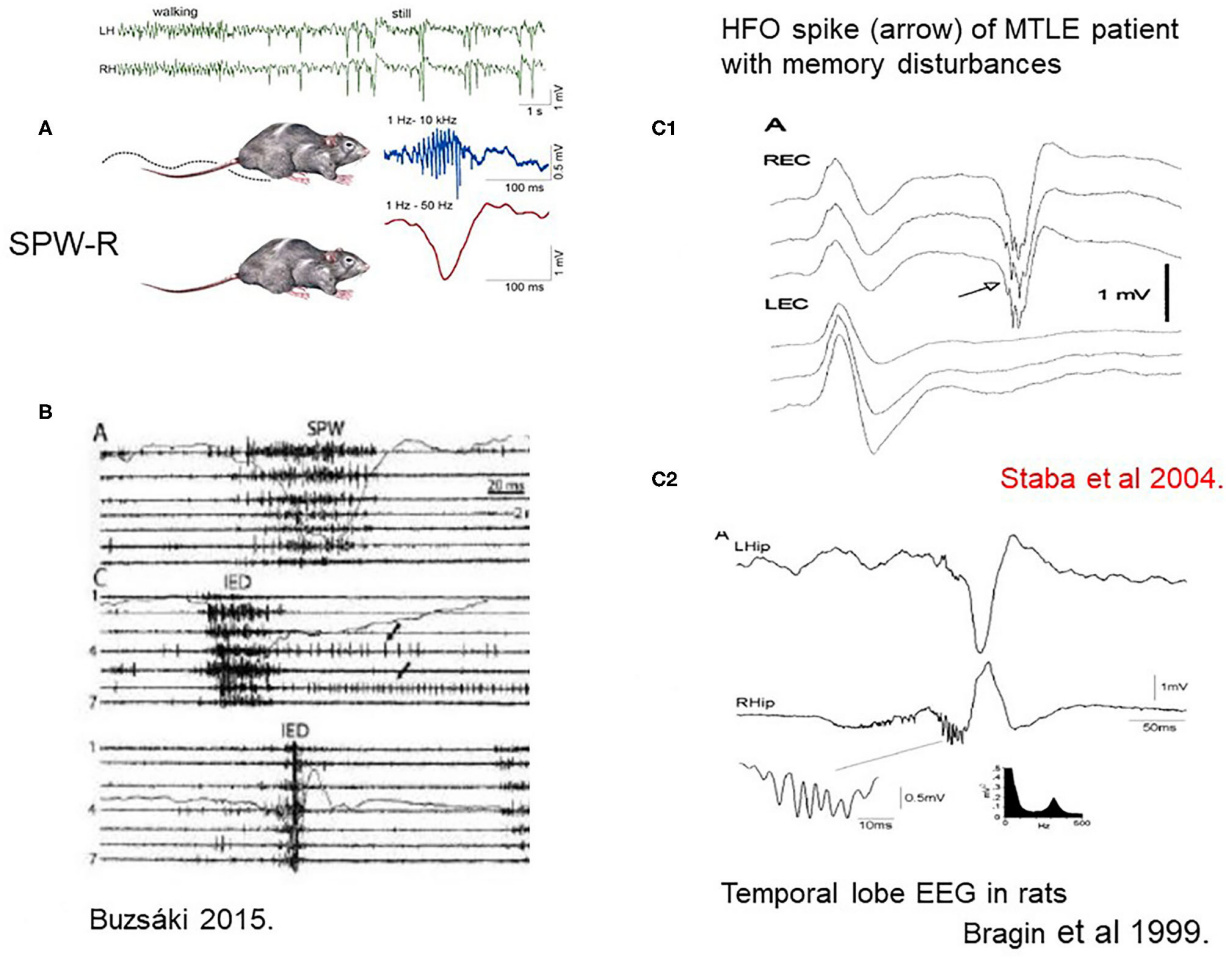
**(A)** Sharp-wave-ripples occur during quiet wakefulness and sleep [upper two EEG channels right [after (7)]. **(B)** Comparison of sharp waves (SPW) and interictal epileptic discharges (IEDs) in the intact hippocampus and after disconnection from its subcortical connections by fimbria-fornix lesion. Channels (1–7) represent different locations along the axis of the hippocampus. See tighter synchrony of population bursts and larger amplitude of the field responses during IEDs]. The shallow line shows the field potentials parallel with the unit discharges [After (7)]. **(C1)** Human MTLE spike and coupled ripple (32). **(C2)** Bragin et al. (33) in rats ().

During quiet wakefulness and NREM sleep, the hippocampo-neocortical network takes active parts in encoding, maintaining and consolidating memory traces with the essential involvement of SPW-Rs. (17). SPW-R is an intensive synchronous excitatory event close to epileptic-level excitation, explaining the high epilepsy-proneness of the hippocampus clear (7) (Figure 2). The epileptic variant of SPW-R is the epileptic spike-pathological ripple complex. Buzsáki described these two kinds of patterns in the hippocampus: the normal SPW-Rs involved in memory processing, and a pathological population spike with similar parameters There are just quantitative differences between them, affecting duration (spikes are shorter), voltage (spikes are higher) and synchrony (pathologic ripples are more synchronous). SPW-Rs' amplitude never exceed 3 μV, and its duration is 30–150 ms. While SPW-Rs couple with 80–200 Hz ripples, the epileptic spikes link with higher frequency ones in the 250–500 Hz range.

First Buzsáki (17) described SPW-R in rodents, then Bragin et al. (33) and Staba and Bragin (34) in a kainic acid epilepsy model and humans.

### The Role of NREM Sleep in the Memory System and in Medial Temporal Lobe Epilepsy (MTLE)

NREM sleep enhances the interictal discharges of MTLE. Using intracranial electrodes, we have a more realistic picture about the big, firework-like amount of limbic spiking. Clemens et al. (35) have shown that foramen ovale electrodes display 2–10 times more spikes during sleep than scalp electrodes do.

There is a link between memory disturbances and hippocampal sleep spiking in human MTLE (36, 37). Hippocampal sleep spindles correlate negatively with the number of interictal spikes (38). This inverse relationship of spindles and spikes suggests that hippocampal spikes might diminish the rate of spindles, i.e., normal spindles transform to spikes. SPW-Rs' conversion to spikes and high frequency (pathologic) ripples was recently evidenced in a rat kindling model (39).

Shatskikh et al. (40) elicited artificial IEDs stimulating the ventral hippocampal commissure and the CA1 region. The evoked discharges resembled to naturally occurring IEDs in epileptic rats and cognitive tests revealed an important cognitive impairment of these animals.

Thus, IEDs might compromise the memory of MTLE patients in at least two ways. (A) Daytime spikes doing as meaningless memory traces deplete synaptic capacity hindering the elaboration of normal memory traces (a hypothesis); (B) IEDs in sleep are consistent with unserviceable ghost images of SPW-Rs that cannot exert memory-processing (39, 41). Thus, in addition to HS, the epileptic transformation of normal hippocampal EEG patterns may impair the memory of MTLE patients.

## Summary

We suggest that essentially, MTLE is the epilepsy of the declarative memory system. The years' lasting epileptic evolution due to an early hippocampal damage rewires the synaptic structure and connectivity network. SPW-Rs transform to spikes with HFO of 200–500 Hz. Homeostatic plasticity normally recovering synaptic balance, shifts toward pathologically exaggerated excitation, leading to chronic epilepsy. IEDs that do not carry relevant information obstruct memory formation and consolidation causing memory disturbances. NREM sleep plays important parts.

## Absence Epilepsy (AE) is the Epilepsy of the NREM Sleep-Promoting System. Transition to Juvenile Myoclonic Epilepsy (JME)

Absences are the most prevalent epileptic seizure types of childhood and young adulthood. The ~3 Hz bilateral spike-wave paroxysms (SW) make their essential EEG-feature. In addition to childhood AE, absences occur in other generalized epilepsies as juvenile absence epilepsy, JME and eyelid myoclonia with absences (Jeavons syndrome) as well.

Additional clinical features and underlying genetic changes discriminate these conditions from each other, however, the electro-clinical syndrome and pathomechanism of absences is likely the same in each one.

### The Relationship of the Corticothalamic System and Absences With Bilateral Spike-Wave Paroxysms

The corticothalamic system was associated for long to the pathomechanism of absences and spike-wave paroxysms (SW) (42).

Gloor from the Montreal Neurological Institute was a pioneer advancing a systematic approach after a long fruitless debate about the cortical or subcortical origin of the pattern. He drew attention to the corticothalamic system (named by him cortico-reticular) and raised the role of the sleep system (43). He proposed that SW emerged from the same circuit normally producing sleep spindles, SW being the epileptic variants of spindles. This idea had far-reaching fertilizing effect on research. Kostopoulos (44) showed that sleep spindles transformed to SW when he placed Penicillin (a GABA-A antagonist) on the cortical surface of cats, however, the number of spindles has not been shown to decrease with the increase of SW.

**Figure 3:**
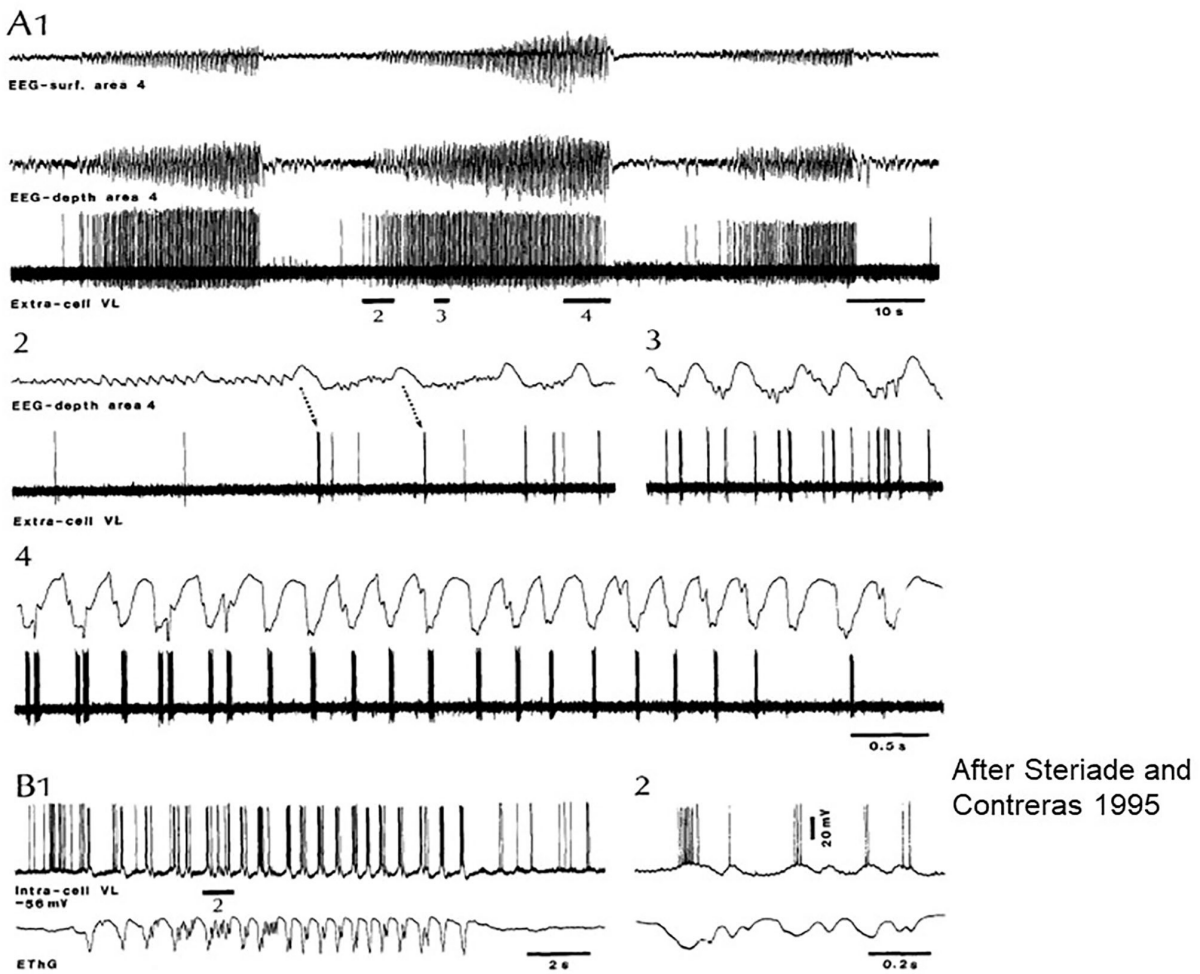
Paroxysmal episode druing sleep induced by Ketamine-Xylazine. Recorded by surface and depth EEG from precruciate area 4 and an extracellular ventrolateral (VL) corticothalamic (TC) neuron in cat. **(A1)** Three paroxysmal episodes separated by 40–45 s. **(A2–4)** The middle one of the episodes: positive cortical waves followed by spike bursts in the VL initiate the paroxysm. (B) Intracellular recording of a rostral VL cell with SW activity at 2 Hz. **(B1,2)** Show the close relation between depolarization and field negativities (after Steriade and Contreras).

Steriade and Contreras (45) revealed intermittent transitions of normal NREM sleep patterns to SW in cats. They showed that “SW originate in the neocortex and are disseminated through mono-oligo- and multi-synaptic intracortical circuits, before they spread to the reticular nucleus of the thalamus (nRE) and exhibit generalized features.” (Figure 3). They supported the cortical origin by several ablation experiments, and stressed the focal to widespread propagation dynamics of seizures. Meeren et al. (46, 47) confirmed the cortical origin in humans and a rat genetic model later.

During SW, the cells of the thalamic reticular nucleus (nRE) discharge a spike-burst while the corticothalamic (TC) neurons display inhibitory postsynaptic potentials (15). This inhibition may lead to hypersynchronization in the form of SW. The key mechanism may be the mutual inhibition between nRE neurons, regulating the level of output inhibition exerted on thalamic relay cells. Simultaneous recording of nRE neurons with TC cells revealed that the rate of corticothalamic inhibitory postsynaptic potentials was the same as nRE cells' spike bursts. Due to nRE neuron's diffuse projections to dorsal thalamic territories, a wide thalamic inhibitory synchronization came off. The GABAergic inhibition coming from nRE resulted in a long-lasting inhibition of TC neurons, obstructing the corticothalamic communication during SW-absences (15). Thus, using the same circuit as spindles do, GABAergic nRE neurons participate in the cortical generation of SW.

Several factors influence nRE neurons' excitability, but the most likely one might be the excitatory input originating from the cortex transforming spindles to SW. Ample variations are possible via the up- and downregulation of excitatory/inhibitory balance in cortical, thalamic, and reticular neurons, including pathways from the brainstem and the cortex. The same distortion of functions resulting in SW can originate from different points of the network.

In an interesting paper dedicated to mechanisms by which epilepsy “hijacks” certain sleep-related circuits^1^, Beenhakker and Huguenard (11) introduced the idea of epilepsies conquering physiologic systems.

### Neuronal Plasticity in the Corticothalamic Network and Its Derailment Toward Epilepsy in NREM Sleep

Steriade and Timofejev (48), applying experimental extra- and intracellular electrode-placement combinations, explored the plastic properties of the corticothalamic network. They used the augmenting response known for long time (49). Single stimuli applied to the pathways ascending to the thalamus or to corticothalamic projections evoked a series of augmenting, incremental responses on the thalamic and cortical levels. This response has very similar parameters as spindles have, therefore, it models sleep spindle oscillations. Both the augmenting responses and spindles cause plastic changes in thalamic and cortical neurons outlasting the stimulation. The patterns display the highest amplitudes during slow wave sleep, while arousal disrupts them.

Slow wave and spindle oscillation dynamics make the corticothalamic circuit prone for SW responses.

### Absence Epilepsy (AE) and Juvenile Myoclonic Epilepsy (JME): Two Members of a Spectrum

Although AE is considered as an age-dependent condition typically disappearing by adolescence, only 58–65% of patients remain symptom free (50, 51). Fifteen to 44% of patients with pure AE progress to JME (50). Seventy percent of JME cases start at age 11–20 years or in young adulthood. There is a saying, “where absence epilepsy decreases, JME starts.” Since the nineties several novel features of JME came to light (52). It has seceded from the idiopathic generalized epilepsy group, which is not a tenable construction any more. At the same time, the sleep-link remained an essential feature, JME seizures appearing closer to waking vs. absences closer to N2 sleep.

The bilateral synchronous short polyspike-wave paroxysms associate with cyclic alternating pattern (CAP) A, especially in high homeostatic pressure periods similar to classic SW (53). The recently noticed photosensitivity (14, 54) brings JME close to reflex-epilepsies. Frontal connectivity changes (55) and prominent behavioral issues (56) point to the extension of the epileptic network to the frontal lobe.

A typical feature of JME is the system-specific increase of motor excitability (14, 57) manifested by myoclonic jerks. Rare generalized tonic-clonic seizures, multiple spikes on EEG and an enhanced responsivity to transcranial magnetic stimulation (paralleling homeostatic regulation) (58) confirm the augmentation of excitability compared to the AE period. The persistence of absences in 15–40% supports the continuity of the two syndromes.

### How Vigilance Level-Changes Across the Sleep/Wake Cycle Influence the Release of Absences?

**Figure 4:**
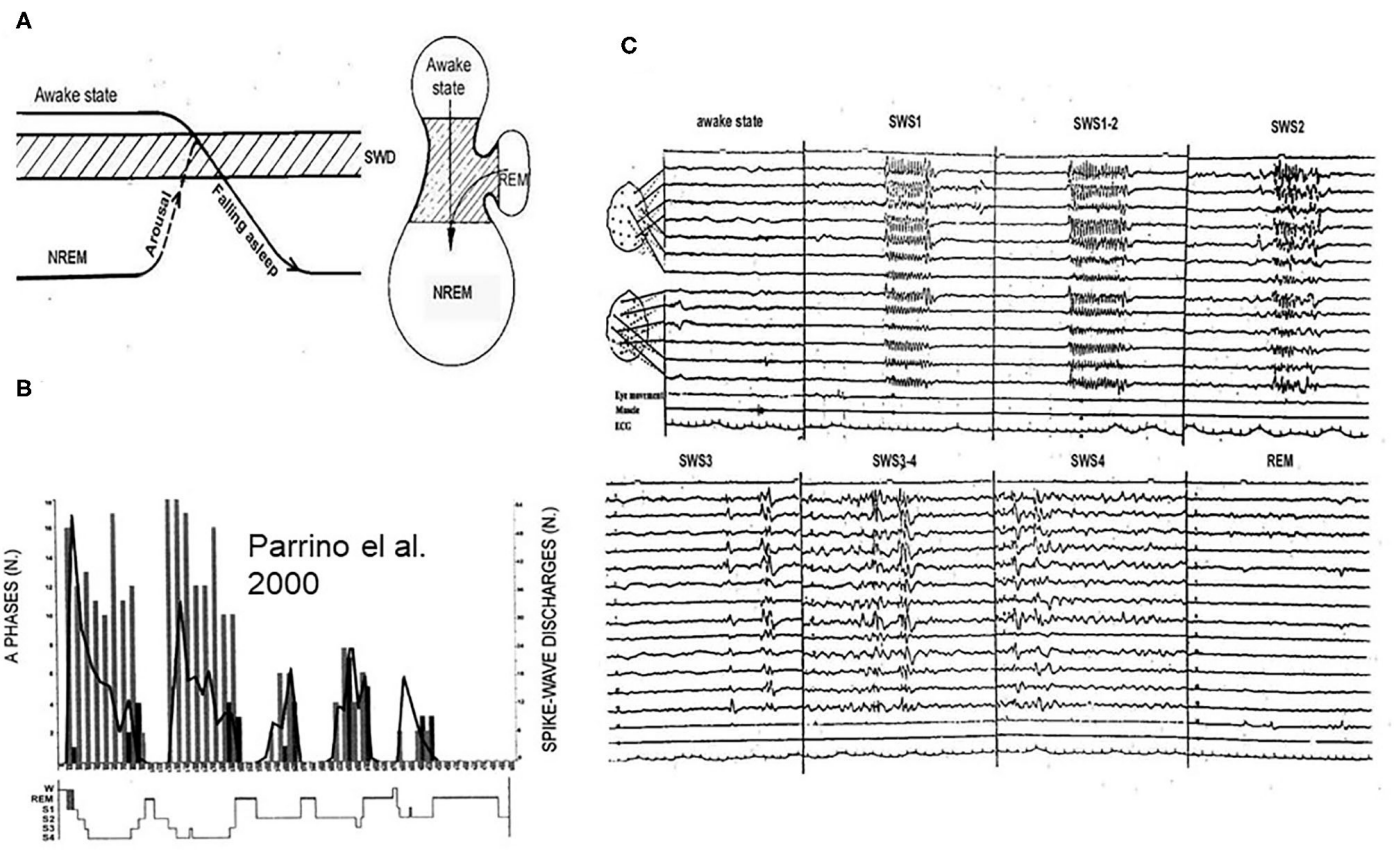
**(A)** The vigilance dependence of absences. Left: Arriving to the critical vigilance state (hatches) from awake sate or from deeper sleep, AE prone patients display absences. Right: the jar- like formation represents the states of existence: lower part: NREM sleep, upper part: awake state, and handle of the jar is REM sleep. The critical zone for absences is in-between. **(B)** Night sleep of a typical absence patient. The perpendicular columns represent cyclic alternating pattern (CAP) A1 phases (phasic slow waves), the graph shows the number of spike-wake seizures: decreasing from evening to morning. **(C)** The distribution of absences across the sleep cycles. During slow wave sleep phase 1 (SWS1) and phase 2 (SWS2) synchronized spike-wave runs (absences) occur, during SWS3 spike wave paroxysms cease, scattered IEDs display (mainly individual spike-waves). During REM sleep, there is no spike wave discharge.

SW occur in a certain vigilance window called “critical vigilance level” for absences (59). These periods are the shifts to-, and light NREM sleep stages until N2 (60–62) as well as other transitional periods (15, 63–65). Arousing stimuli stop absences (66) and they do not turn up in REM sleep. However, if NREM sleep or waking EEG-patterns fragment REM sleep, SW may appear (59) (Figure 4).

Based on clinical observations, absences were considered awake events however, their close relation with vigilance-level decreases even within the awake domain has become clear (65, 67). When apparent arousal influences induce absences, a closer analysis may reveal the actual link with reactive sleep-like anti-arousal responses (68). The vigilance level-dependence explains the distribution throughout the 24-h sleep/wake cycle.

### Sleep Microstructural Changes and SW

During sleep, there are peculiar transitional states of vigilance with frequent bidirectional oscillations toward sleep or arousal. The homeostatic pressure determines the types of phasic responses to arousing stimuli. When the homeostatic pressure is high, sleep-like slow waves (CAP A1) appeare, and when it was low, a desynchronized arousal-like or mixed response occur (CAP A3/A2). There is a strong positive correlation between CAP A1 and SW (69–72).

Applying sensory stimulation to manipulate these oscillations, we have shown that more SW occur during fluctuations toward NREM sleep compared to shifts toward the waking or REM sleep (59, 73) (doctoral thesis). Sensory stimuli induced vigilance-fluctuations and elevated the number of SW. Terzano (69), showed frequents SW during CAP phases. In JME patients (70), the spiking rate was significantly higher in CAP A1 compared to non-CAP while CAP B inhibited SW. Seventy percent of SW occurred in CAP A1, 24% in A2, and 6% in A3 (74). In other words, SW were the most prevalent in the first cycles, declining later simultaneously with the decay of the delta power from evening to morning. On the descending slopes of sleep dominated by subtypes A1, the CAP-related activation of SW was triple compared to the ascending slopes containing more A2 and A3 events.

In 13 children with AE, most generalized discharges occurred during sleep-fluctuations, especially during shifts from CAP B to A, i.e., SW occurred during shifts toward NREM sleep (72).

Thus, absences prefer low and shifting-vigilance periods within the realm of superficial NREM sleep (8) and they are associated with CAP A1 phases (slow waves, reflecting shifts toward NREM sleep). The activating effect of sleep deprivation and sleep itself is likely due to the same mechanism (71).

### Is Absence Epilepsy the Reflex Epilepsy of NREM Sleep Promoting System?

The triggers of classic reflex seizures are specific external/internal or cognitive stimuli putting into motion an epilepsy-prone system. This, potentially normal activation may switch to an epileptic working mode: a seizure. This concept conceives the stimulus as seizure trigger, but looking into this closer, the activation of the system carrying pre-existing hyper-excitability actually promotes the seizures. The epileptic hyperexcitability is a sine qua non. Regarding absences, the activation of the sleep-promoting system may be a “reflex” seizure trigger (75, 76).

## Summary

Going into NREM sleep, the working mode of the corticothalamic system changes. Recurrent packages of alternating inhibitory-excitatory cycles develop while building up the characteristic burst-firing cellular activity in the corticothalamic system, represented by slow waves and spindling on EEG.

NREM sleep corticothalamic burst-firing mode normally producing spindles is apt to transform to SW-absences as declared by Gloor 42 years long ago. Genetic factors, input changes, and neurotransmitter effects make individuals prone to this transformation. The key mechanism seems to be the mutual inhibition between nRE neurons, regulating the level of the output inhibition exerted on thalamic relay cells. If the output inhibition from the nRE is high (intracellular inhibition low) the thalamic relay cell inhibition will be more effective, leading to hypersynchronization in the form of SW.

In this paragraph, we accumulated evidence supporting the existence of such transformation. There are several factors facilitating it: high homeostatic pressure, pronounced vigilance oscillations in the critical transitional vigilance zone; and shifts toward superficial NREM sleep characterized by sleep-like slow waves (CAP A1).

The studies of Steriade and Timofejev show that the corticothalamic system during sleep is beweaponed by homeostatic synaptic plasticity in the form of augmenting potentials. When this plasticity comes into effect, the increase of excitability may reach an epileptic level and lead to seizures and chronic epilepsy.

We propose that AE with the conjoined 2–4 Hz SW may be the epilepsy of the sleep promoting system contrasting the epilepsy of the antagonistic arousing system—nocturnal frontal lobe epilepsy.

## Nocturnal Frontal Lobe Epilepsy (NFLE) as the Epilepsy of the Cholinergic Arousal System in NREM Sleep

### The Macro- and Microstructure of NREM Sleep and the Arousal Phenomenon

The possibility of arousal and reactivity to sensory stimuli are essential features of sleep differentiating it from coma, keeping the sleeper in contact with the environment. Microarousals - arousals without awakening - serve the reversibility of sleep and carry an alarm function in danger.

Microarousals used to be considered harmful perturbations destabilizing sleep; however, they turned out to have a sleep-regulatory (8, 77) and a safeguarding function. They make inherent parts of the dynamic sleep structure organized under the CAP system (78).

The notions about the nature of sleep change continuously. Those data pointing to local/regional sleep and waking states have challenged the “global” sleep idea (79, 80). In certain conditions affecting sleep, the normal mosaic like local co-occurrence of vigilance states turn pathologic. For example, the defining feature of arousal parasomnias is state dissociation, where waking and sleeping behaviors happen together.

### NREM Arousal Parasomnias (AP)

The waking state, NREM and REM sleep may appear locally, in circumscribed small areas of the brain. Local sleeping/waking are normal phenomena but there are pathologic forms as well (81), the most obvious ones being parasomnias.

Based on the hosting sleep states, REM and NREM parasomnias are distinguished, the NREM variants also called “arousal parasomnias.” NREM parasomnias include confusional arousals, sleepwalking and sleep terror. They favor the childhood and have a genetic origin (82).

In sleep terror episodes, there is an abrupt motor/autonomic arousal from NREM sleep, while sleep persists in a circumscribed area involved in cognition. Two recent publications have provided congruent evidence for such dissociated conditions in AP (83, 84). Since the partially sleeping brain does not perceive the actual situation while the strong autonomic excitation of arousal signalizes danger, the patient cannot recognize or remember the typical autonomic and motor agitation featuring the condition.

### The Epileptic Counterpart of Arousal Parasomnias

The counterpart of arousal parasomnias is frontal lobe epilepsy with an adventurous history reflected by changing names. Its first name “nocturnal paroxysmal dystonia” expressed the belief of a sleep-related movement disorder (85). The second name “idiopathic nocturnal frontal lobe epilepsy” (NFLE) expressed the idea of an epileptic disorder (86–88) and a recent consensus conference renamed it again to “sleep related hypermotor epilepsy” (SHE) (89). This concept incorporated any forms of sleep related epilepsies with hypermotor seizures, irrespectively from the variable types of movements related to the ictal zones within the large frontal lobe.

Most NFLE seizures are cryptogenic, while in a minority of patients, there are mutations in the nicotinic acetylcholine receptor (nAChR) gene subunits. These patients make the group of autosomal dominant nocturnal frontal lobe epilepsy (ADNFLE) (90) and there are newly recognized additional genetic causes, too (91). The electro-clinical symptoms do not discriminate autosomal dominant nocturnal frontal lobe epilepsy (ADNFLE) from the rest of NFLE (87).

The seizure-symptoms of NFLE are consistent with the spectrum of variable degree arousals: simple, seemingly spontaneous fragments of microarousal-related behaviors, arousal, frenetic panic and hypermotor agitation, even movement storms with autonomic and emotional expressions of alarm and consistent EEG/EMG and behavioral signs. The description is “hypermotor seizures” (92, 93).

SPECT studies revealed the same ictal hyperperfusion-zones during seizures of one patient, but the region of ictal hyperperfusion varied in different patients even within the same family (83).

During the waking state, IEDs hardly occur, and they appear seldom during sleep, too, occurring in not more than 50% of the patients. Fronto-basal discharges may project to the anterior temporal leads and fronto-medial ones to the midline electrodes, making proper localization hard. A typical ictal EEG pattern is rare, too. In about half of seizures, some kind of ictal electric changes as rhythmic theta, delta or flattening appears. The extended group of SHE involves symptomatic cases including ones with cortical dysplasia as well. The highly active IEDs featuring this group seem to constitute a distinct population, at least from point of view EEG. Many patients respond well to carbamazepine; about 30% is treatment-resistant (86, 87). There is some anecdotal evidence for the beneficial effect of acetazolamide (94), nicotine patch and fenofibrate (95).

### Is NFLE Associated to the Disorder of the Cholinergic Arousal System?

Acetylcholine (Ach) plays an important role in the activation of the frontal cortex during arousals (96, 97). The thalamus and the cortex are rich in cholinergic fibers, originating from the basal nucleus of Meynert providing a robust cholinergic input. Both the trans-synaptic and non-synaptic release of Ach is significant (98). The mutant nAChR genes in autosomal nocturnal frontal lobe epilepsy (ADNFLE) might compromise the cholinergic arousal system. There are mutant receptor genes in the thalamus, the mesencephalic tegmentum belonging to the ascending arousal system and in the frontal cortex (99) and mutant nicotinic acetylcholine receptors (NAChR) in the mesencephalon, an adjacent part of the diencephalon and the cerebellum (100, 101). They can cause hyper-activation (gain of function) of the frontal cortex mediated by corticothalamic connections (100, 101).

The interpretation of seizures with agitated escape-reaction, panic-like behavior and hypermotor features is dubious. These behavioral patterns traditionally belong to the frontal lobe.

The seizure symptoms may be consistent with an epileptic activation of automatic movement “conserves,” normally suppressed in subcortical generators. When the descending frontal liberation ceases due to a seizure, those atavistic animal or infant behaviors may disengage (102).

Another concept is that the movements and autonomic signs are alarm-responses evoked by an exaggerated arousal. The prominent arousal features in the smaller “simple arousal” seizures support this presumption.

We propose that NFLE seizures are consistent with exaggerated and partial arousals: pathological motor, emotional and autonomic arousal-behaviors co-occur with the frontal dorsolateral cortex (carrying cognitive functions) remaining in sleep.

#### AP and NFLE Share the Symptomatology of Arousal Disorder

There is a parallelism between NFLE and AP. Derry et al. (93) compared the semiology of 63 idiopathic NFLE patients' seizures with 57 AP episodes based on vide-EEG monitoring. They identified three fundamental patterns, but most events contained a composite of more than one.

The basic patterns in both groups were the followings: (1) Simple arousal behavior (92%): eye opening, head elevation, staring, face rubbing, yawning, stretching, moaning, and mumbling. (2) Non-agitated motor behavior (72%): sitting forward, manipulation of nearby objects, orientation. Standing or walking were rare in the patients mounted with electrodes. Patients had a passive face or looked perplexed. Coherent speech-fragments frequently appeared. (3) Distressed emotional behavior (51%): signs of fear and anguish marked by facial expression and speech content. Patients sat or stood up, screamed, and behaved franticly. Attempts of restrain evoked an aggressive response. The comparison of NFLE and AP in the Derry study showed that 3/4 of AP events began with an arousal and in 2/3 of them, violent symptoms followed.

Arousal behaviors preceded half of NFLE seizures, too, while the other half started abruptly, indistinguishably from the non-heralded 1/4 of parasomnia episodes. Tachycardia frequently occurred in both groups. An external or internal stimulus triggered more than one third of AP episodes, while <1/10 of NFLE seizures had a trigger. In the NFLE group environmental interactions occurred just in 11% of seizures and frenetic, non-interactive but coherent speech was rare. Twenty-five percent of AP episodes terminated in wakefulness, while 88% of seizures awakened the patients.

We can draw several lessons from this study. NFLE seizures and parasomnia events were astonishingly similar and followed a parallel severity order, too. A frequent pattern was an autonomic arousal in both groups; culminating in the 3rd degree behavioral pattern (according to Derry's classification), called hypermotor seizure in NFLE, sleep terror in AP. A trigger was more frequent in AP events compared to seizures. After this study, Broughton's classification of AP to the distinct groups of confusional arousals, sleepwalking/somnambulism and sleep terrors seems outworn, a continual symptom-spectrum within a unified AP category, fits better. The symptomatic similarity of seizures and AP events suggests a shared mechanism of arousal disorder. The meanwhile established genetic links (103) support AP's and NFLE's kinship. Another consequence of the Derry study is the recognition that the usual “hypermotor” description of NFLE seizures does not cover the symptom-spectrum from fragments of arousals through non-agitated motor-, to serious alarm behaviors. In addition to the quantitative gradation-differences, frontal lobe seizures are qualitatively heterogeneous, too, due to their variable localization in the large frontal cortex and including those cases with secondary frontal spread from extra-frontal seizure-onset zones.

### Common Sleep Physiopathology, State Dissociation, and Genetic Aspects in AP and FNLE

#### Sleep Relations

Both NFLE seizures and AP episodes link to sleep micro-arousals (104). Several NFLE seizures emerge during one night, in contrast to just one or two AP events. Both conditions manifest in the first or second sleep cycle accumulating in the first one. The frequency of episodes decreases during the night, paralleling the homeostatic decay both in AP an in NFLE.

#### Common Genetic Background

There are genetic origins underlying ADNFLE delineating in the minority of the NFLE patients a mutation in the nicotinic acetylcholine receptor (NAChR) gene subunits (90, 91). There is a conspicuous familial accumulation of parasomnias (105). Twin studies have shown higher concordance for sleepwalking in monozygotic than dizygotic twins (100). Based on the study of a four-generation family, Licis et al. (106) described the first genetic locus for sleepwalking at chromosome 20q12-q13.12 and suggested an autosomal dominant trait with reduced penetrance.

In a survey of 100 NFLE cases, Provini et al. (87) showed that more than 1/3 of patients had a family history for epilepsy and 1/3 of them had had parasomnias.

Bisulli et al. (103) compared 33 NFLE patients and their 200 relatives with 31 age, sex, education, and residence area-matched controls and 194 relatives, for the rate of arousal parasomnias. The lifetime prevalence of APs was more frequent in NFLE patients' relatives compared to the relatives of the controls. APs and bruxism were more frequent among NFLE patients than controls. This association was significant for sleepwalking and showed only a tendency in other arousal parasomnias. Some childhood parasomnias swapped to NFLE later in life (87).

#### State Dissociation With and Without Epilepsy

The electric and behavioral co-occurrence of different local vigilance states- establishes the pathological state-dissociations in AP. In cases of an epileptic transformation, the fundamental situation is similar. The essential feature of epileptic networks is that they exaggerate the functions of the affected physiological systems (107, 108). The normal activation of the network may progress to the epileptic exaggeration of the network that anchors it.

The best candidate for a hosting functional system of NFLE is the frontal cholinergic arousal system, serving the maintenance of wakefulness and targeted attention in the daytime and producing micro-arousals during sleep. In ADNFLE and possibly in the larger family of NFLE, the cholinergic arousal system is sensitized by a mutation of Ach receptors lowering arousal-threshold in NREM sleep (81). The symptomatology of NFLE seizures is consistent with arousals without cognition or awareness - a dissociated state.

## Summary

The recognition of state-dissociation may contribute to the understanding of AP and NFLE. Both conditions show similar symptomatology and a shared genetic background. AP is more time limited, while epilepsy lasts longer. It is striking that there are two related genetic conditions, non-epileptic and epileptic; both strongly associating to the arousal system's hyper-function and NREM sleep dissociation states with similar symptoms and an overlapping time-window. The data about the transition of APs to NFLE are not convincing, although NFLE starts typically later than AP. The cholinergic origin is clear in the epileptic group unlike APs, but the similarity of clinical symptoms, the link to NREM sleep microarousals and the individual and familial overlap of the two conditions, support a common cholinergic origin.

## Idiopathic Focal Childhood Epilepsies (IFCE) and Their Transformation to Epileptic Encephalopathies

**Figure 5:**
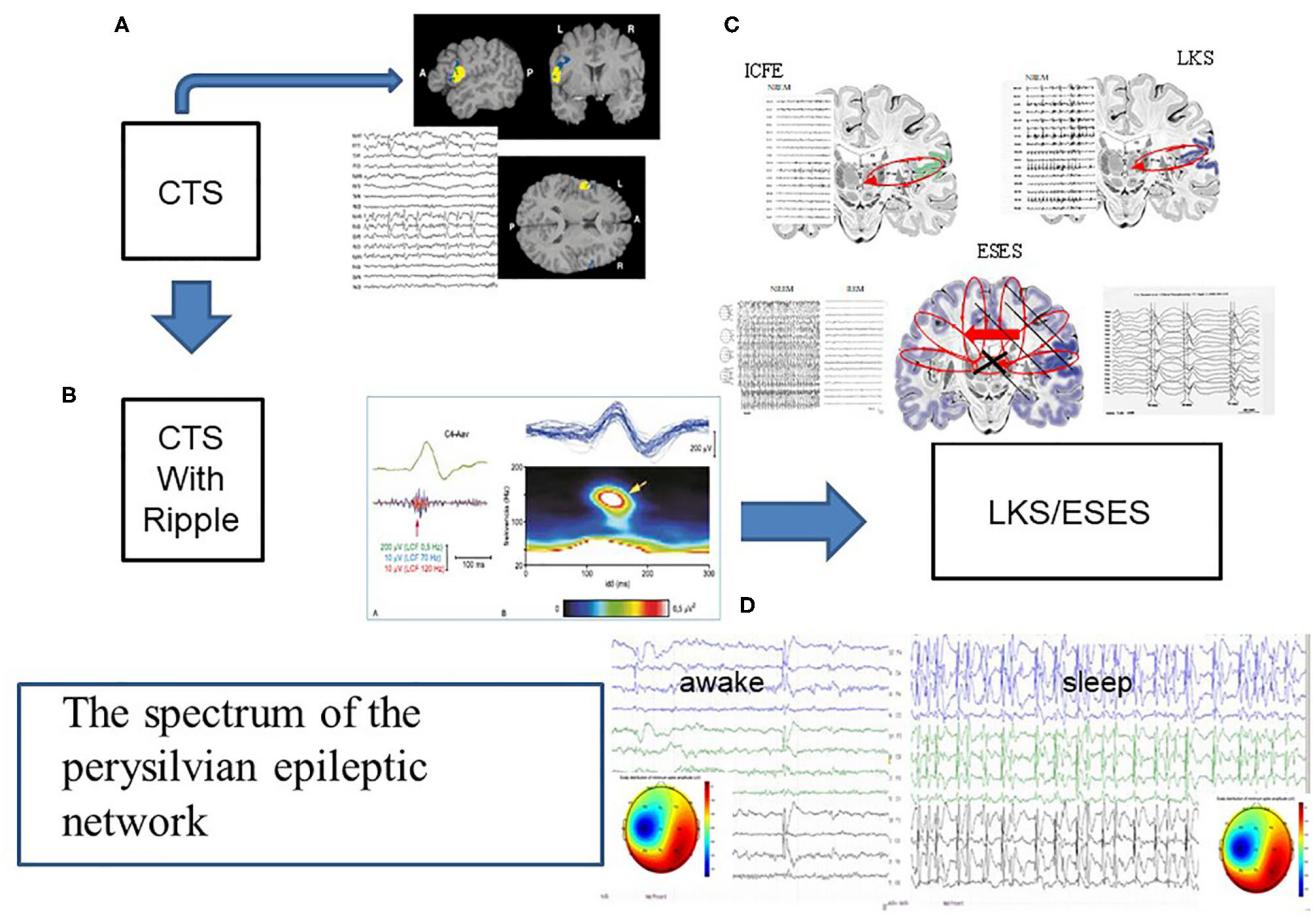
The spectrum of the perisylvian epileptic network. **(A)** The classic centrotemporal spike (CTS) [endophenotype of idiopathic focal childhood epilepsies (ICFE)- Focal appearance by fMRI, and the EEG pattern, **(B)** CTS with ripple in electrode C4, representing the ICFE syndromes (Rolandic epilepsy-RE and Panaiyotopoulos syndrome -PS). **(C)** Schematic display of ICFE, LKS and ESES. ICFE circuit in anterior pesylvian area, LKS in the posterior perisylvian area, and ESES over both hemispheres with a leading one. On the left insert: hemispheric delay in the transmission of the discharges]. **(D)** Transformed encephalopathic syndromes (ESES/LKS). During awake state IEDS; during sleep continuous discharges. The amplitude map of discharges of singular or serial IEDs both in waking state and sleep, show similar uni-hemispheric pattern.

The third group of epilepsies – idiopathic focal childhood epilepsies (IFCE) and their transition to electrical status epilepticus in sleep (ESES) and Landau-Kleffner Syndrome (LKS, acquired epileptic aphasia) make a spectrum-disorder, too (Figure 5). This group constitutes a large mass of childhood epilepsies with a prevalence of 15–20% in children younger than 15 (109, 110).

Raising several crucial issues of epileptology, these epilepsies represent important practical and heuristic challenges.

We will describe the characteristic features of ICFE based on the two core-syndromes Rolandic epilepsy (RE, benign centrotemporal epilepsy) and Panayitopoulos syndrome^2^ (PS) and their transformation (malignization) to the encephalopathic variants electrical status epilepticus in sleep (ESES) and Landau-Kleffner syndrome (LKS). The proportion of transforming cases is unclear.

### The Human Perisylvian Network (PN)

The PN surrounding the Sylvian fissure is strongly engaged in communication and cognition. Since the PN hosts speaking and understanding speech, as well as reading/writing/calculating, we coin it with a new functional name, “human communication network.”

#### Regional Distribution of Communication Functions in PN

The PN harbors important human-specific cognitive functions. The frontal operculum, the first temporal convolution and the angular gyrus around the end of the Sylvian fissure are regions mainly associated with speech and reading. PN areas strongly interlink forming a broader network called associative cortical areas. They have rich connections with thalamic structures (113, 114) revealing why thalamic lesions may lead to language symptoms. The recent progress of functional neuroimaging allowed to elucidate the roles of PN regions in speech and other communication functions as well as to better understand the links of Rolandic epilepsy with motor and language networks (115–118).

PN involves the arcuate pathway connecting the Broca and Wernicke areas in the left hemisphere. These pathways pass through the inferior parietal cortex running parallel and lateral to the arcuate fasciculus, making direct and indirect connections. The anterior segment of the indirect pathway connects the Broca region with the inferior parietal lobe, while its posterior segment connects the inferior parietal lobe to the Wernicke area (119). The PNs of the two sides serve different functions: language and praxis in the left hemisphere and spatial orientation in the right (120).

The perisylvian pathways have variable lateralization; a bilateral representation may be favorable. The language representation is bilateral and symmetrical in 17.5% of normal humans; more in females (119).

### The Core Syndromes of Perisylvian Epileptic Network: Idiopathic Focal Childhood Epilepsies (IFCE): Rolandic Epilepsy (RE, Centrotemporal Epilepsy) and Panayiotopoulos Syndrome (PS)

IFCEs are time-limited, age-dependent conditions of age 3–10 years, fading away by age 15–16. Based on 30 years clinical observations and research, Panayiotopoulos et al. (121) characterized them: “All these conditions, established core syndromes of idiopathic focal childhood epilepsies (RE, PS and Gastaut type occipital epilepsy) and the newly described variants may be linked together in a broad, age-related and age-limited, benign childhood seizure susceptibility syndrome, which may be genetically determined.” Panayiotopoulos syndrome (122–124), constitutes about 13% of epilepsies under 6 years. A consensus report (125) characterize it as “benign, age related focal type of epilepsy developing in the early and mid-childhood”.

#### Seizures

In Rolandic epilepsy, the typical ictal symptoms are facial sensory- and oropharyngo-laryngeal motor; speech arrest and salivation. These symptoms suggest an anterior PN and anterior intra-Sylvian network (126). Very rare secondary generalized tonic-clonic seizures occur mainly from sleep.

PS seizures are less localized, two third of them starting in night NREM sleep (less than in RE). They may be longer than 30 min, are not always stereotyped and sometimes aggravate to status epilepticus. Seventy percent present with vomiting (from nausea to retching), color and temperature changes, mydriasis, incontinence, salivation, vehement bowel movements, respiratory and heart rhythm changes, and syncope. Convulsions and atony may happen, too. Eye deviation is infrequent. Despite occipital spiking, seizures do not start from the occipital region, visual hallucinations hardly occur. Seizure symptoms suggest an epileptic central autonomic network (127) involving the insular and medial prefrontal cortex, the amygdala and hypothalamus: the immaturity of the autonomic system might underlie the low seizure threshold (128). The consensus view (125) classifies PS as an autonomic epilepsy.

#### Interictal Epileptiform Discharges (IEDs)

IEDs (Rolandic spikes, centrotemporal spikes, CTS) of RE originate from the face-field of the somatomotor gyrus. Abundant sleep spiking may emerge in clusters and originate from the contralateral side of focal seizures. Independent bilateral discharges are frequent. A slow wave closes the spikes. They have a characteristic frontal positive and temporal negative dipole perpendicular to the Sylvian fissure. A MEG localization study (129) showed that the cortical generators of CTS lie in the precentral motor cortex. The centrotemporal potential fields may shift to the frontal and parietal regions, preferring big cortical fissures (Sylvian, parieto-occipital), suggesting a vulnerability for maturational micro-defects in territories of sophisticated gyral development. In superficial sleep or wakefulness, short generalized spike-paroxysms may appear. Thus, there is polyfocal spiking. During long-term follow-ups, posterior discharges may shift forwards (130, 131) paralleling the change of ictal features (132).

In PS, multiple changing localization, typically occipital, morphologically CTS-like discharges occur.

Clemens and Majoros (133) found the strongest CTS-activation of IFCE during deep slow wave sleep, especially on the descending slopes of the first sleep cycles. Unlike the rest of epilepsies where IEDs couple with the slow waves of CAP A1 (74, 134–138), CTS rather associate with spindles^3^; also in Landau-Kleffner syndrome (LKS). Kobayashi et al. (139) reported that CTS link with HFO of 93.8–150.3 Hz (mean 126.2–136 Hz). HFO was significantly more prevalent near seizures.

The old name: *benign* centrotemporal epilepsy, may have originated from the belief that RE did not cause any cognitive loss and it was time-limited. However, several studies including an ILAE position paper (140) reported on various related behavioral and cognitive deficits in writing, verbal expression, comprehension and working memory (141, 142), long-term storage and retrieval (143) as well as language and academic performance (144). The cognitive deficits link the ICFE with their malignant variants electrical status epilepticus in sleep (ESES) and LKS (112).

### The Transition of Idiopathic Focal Childhood Epilepsies to Electrical Status Epilepticus/Landau-Kleffner Syndrome (ESES/LKS)

IFCE and ESES/LKF encephalopathies used to be considered distinct entities, and even LKS and ESES had separate stories based on the regional (LKS) vs. global (ESES) involvement. One of the difficulties unifying these conditions into one spectrum was the supposed big gap between the cognitive outcomes in simple IFCE and the encephalopathic forms. The chaos of terminology^4^ caused additional confusion.

Patry et al. (147) described a “peculiar EEG pattern occurring almost continuously during sleep, characterized by apparently subclinical spike-and-waves, for variable length of time (months to years)” in six children with cognitive deficit. After this first publication, Tassinari et al. (148) introduced the term “encephalopathy related to electrical status epilepticus during slow wave sleep.” They suggested, “The condition of a protracted (years) status epilepticus in sleep can be the factor leading to severe mental deterioration and psychic disturbances.” Initially, ESES seemed seldom (149) but more and more cases were identified. Its pathomechanism remained unclear: more than half of patients had a severe lesional background, and only the rest originated from IFCE.

RE was the first condition recognized to progress to ESES. Dalla Bernardina et al. (150, 151), Lerman and Kivity (152), and Panayiotopoulos (122) broadened the concept to all variants of IFCE as age-dependent transitory idiopathic regional hyperexcitability syndromes underlain by genetic maturational abnormalities (122, 153). We published a very similar view (112) providing evidence for unifying the syndromes of IFCE, LKS, and ESES into one spectrum. Fejerman (154) recognized the “atypical evolution of Rolandic epilepsy,” defined by severe neuropsychological impairments and continuous spike-waves (CSWS) during slow wave sleep. More recently, a retrospective study including 196 self-limited, pharmaco-responsive childhood epilepsy patients with CTS suggested that ~7% of patients progressed to ESES or LKS representing 65% of all “atypical” forms of CTS (155). Others reported that about one-third of ESES cases had a previous diagnosis of IFCE (156, 157), while in their synthetic work, Panayiotopoulos et al. (121) devoted only one page to the malignization treating it as a rare complication (<1%) of IFCE cases. Recent literature shows that ESES/LKS are prevalent conditions (146, 154, 155, 158–169).

Kellermann (170) first documented that patients with acquired epileptic aphasia (LKS) had an extreme activation of spike-and-slow wave discharges during NREM sleep consistent with ESES. Since then, several authors have considered LKS a variant or a subtype of ESES (118, 162, 164, 171–176).

The evolving dysphasia in LKS suggests a circumscribed functional disturbance of the speech-related perisylvian opercular structures in the posterior first temporal convolution, as supported by some success of the Morrell-type surgical interventions performed in these structures (177).

Bilateral EEG discharges occur frequently in both LKS and ESES. The involvement of the dominant PN may lead to dysphasia in LKS while the widespread epileptic dysfunction may cause a more global cognitive loss in ESES, possibly through the corticothalamic system (178).

Interictal cognitive deterioration closely link - likely correlate - with the localization, amount and persistence of sleep-related CTS suggesting the causative role of the epileptic process.

The duration of the period with abnormal sleep determines the degree of cognitive decline (146, 179). No residual deficit remains if ESES-length is <13 months, but the cognitive impairment may be chronic, if it is longer than 1.5 years (157, 176), leaving half of patients with severe impairments (149, 175, 179, 180). While the journal Epileptic Disorders dedicates a special issue (volume 21 June 2019) with several updated papers for ESES, just a few studies provide evidence on the correlation of cognitive impairment with the assumed deficit of sleep slow waves' downscaling (181–184).

In addition to CTS, pathological HFO has an impact on cognitive impairment, too (139, 185, 186), confirming the causative role of IEDs.

### Spectral Features

Carrying their own peculiarities and nature, IFCE syndromes overlap. They have shared genetics, a common pattern of interictal activity - the CTS endophenotype - and the augmenting effect of sleep on the rate and continuity of CTS dictating cognitive loss. The overlap, continuity and mutual transition from RE to PS and vice versa as well as the unidirectional progress of them to LKS/ESES unify these conditions into one spectrum (187).

These data change the concept of PN epilepsies to broad, genetically determined conditions of high cortical excitability and slipping epileptic zones. The epileptic dysfunction affects a widespread network of associative cortices. The corticothalamic system might be the candidate relay station of the epileptic propagation (112).

#### Genetic Aspects

PN epilepsies have been linked to a number of genetic features

- SRPX2 and ELP4 genes' involvement with possible impact on cell motility, migration and adhesion (162);
- Changes in the GRIN2A gene encoding the NMDA receptor NR2A subunit as a major genetic risk factor for IFCE (188).
- Increased copy-number variations in the PN spectrum conditions in the genomic architecture of genes encoding cell adhesion proteins (189);
- Some genetic factors causing channelo-pathies may underlie the development of ESES/LKS (190).

#### The Centrotemporal Spike as an Endophenotype

Endophenotypes are genetically based common modules of phenotypically different disorders like schizophrenia, autism, attention-deficit hyperactivity syndrome and, certain epilepsies (112). CTS is an endophenotype occurring in NREM sleep both in IFCE and non-epileptic individuals (150). It is shared by PN conditions, autism spectrum disorders (191, 192) and ADHD (193–195), but it is much more prevalent in epilepsies. CTS is a peculiar pattern occurring in 2–4% of normal children, too (196, 197). It plays as and augmented acoustic or somatosensory evoked potential in infants (198, 199). It is similar in RE and PS patients, before and after the progress to ESES (156, 157, 200), evidencing the spectral togetherness of those conditions. The potential field of CTS in ESES patients is the same in waking and in sleep (146).

Even without epilepsy, CTS may affect a broad network in NREM sleep. This was shown by the case of a 13-year-old non-epileptic boy with difficulties of reading, writing and calculation. He had right dominant bilateral independent CTS augmented by NREM sleep. fMRI performed in wakefulness showed an increment of the BOLD signal in the bilateral sensory-motor cortex while fMRI in NREM sleep highlighted a much more widespread CTS-related network in the PN and the connected thalamic region [Mirandola et al. (201) in 2013].

In non-epileptic cases there are no ripples associating with CTS. HFO “crown” CTS in epilepsy patients (RE) and there are even more ripples (HFO) on the top of CTS in the malignant forms (185, 186). There are just quantitative differences (amplitude and frequency) discriminating the good and poor outcome (139).

Despite the name “spike,” CTS is actually a sharp wave of 88-ms duration (202). It is electro-morphologically similar to premature infants' delta-brush (203) that are supposed to have an impact on the somatotopic arrangement in the sensory-motor cortex (204, 205).

Concluding these data, CTS is the IED and endophenotype of PN epilepsies. It could represent a local cortical developmental delay and augmented excitability that might regress or develop to epilepsy (122, 153). The quality, synchronization and spread of CTS parallels the clinical condition from good outcome cases to malignant encephalopathy variants (112).

#### Continuity of CTS Dynamics During Sleep and Wakefulness

CTS-rate varies from rare and random (1–3/10 s) to almost continuous and the topography is variable, too. CTS frequently occur bilaterally and independently, posteriorly, in the centro-temporal or occipital regions. Multiple spike foci in one recording are frequent in PS. Singular IEDs may evolve to secondary propagation.

**Figure 6:**
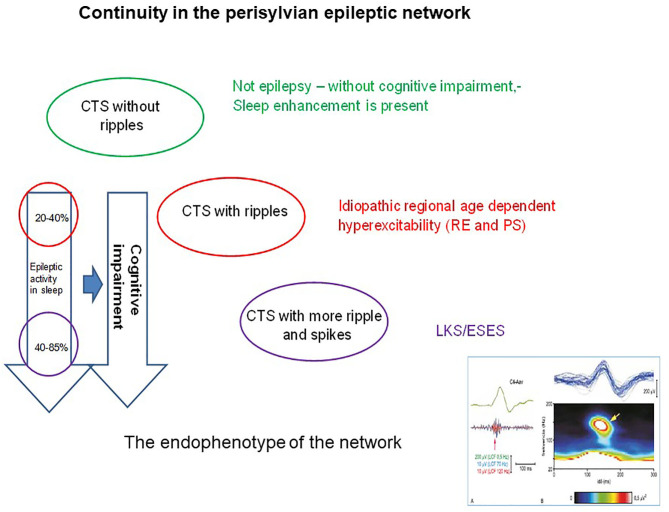
The continuity in the perisylvian epileptic network. The colors indicate the syndromes in the spectrum. Identically colored circles containing the changes in pattern of the common endophenotype (the centrotemporal spike). The perpendicular arrows indicate the degree of cognitive impairment (right arrow) proportional with the degree of spiking (left arrow). The right insert below shows the shared endophenotype (CTS with ripples).

The sleep augmentation of CTS is a typical feature of each IFCE condition (Figure 6). The amplitude of CTS is higher on the descending slopes of sleep cycles where CTS numbers parallel delta waves; both phenomena suggesting a homeostatic regulation of CTS occurrence.

**Figure 7:**
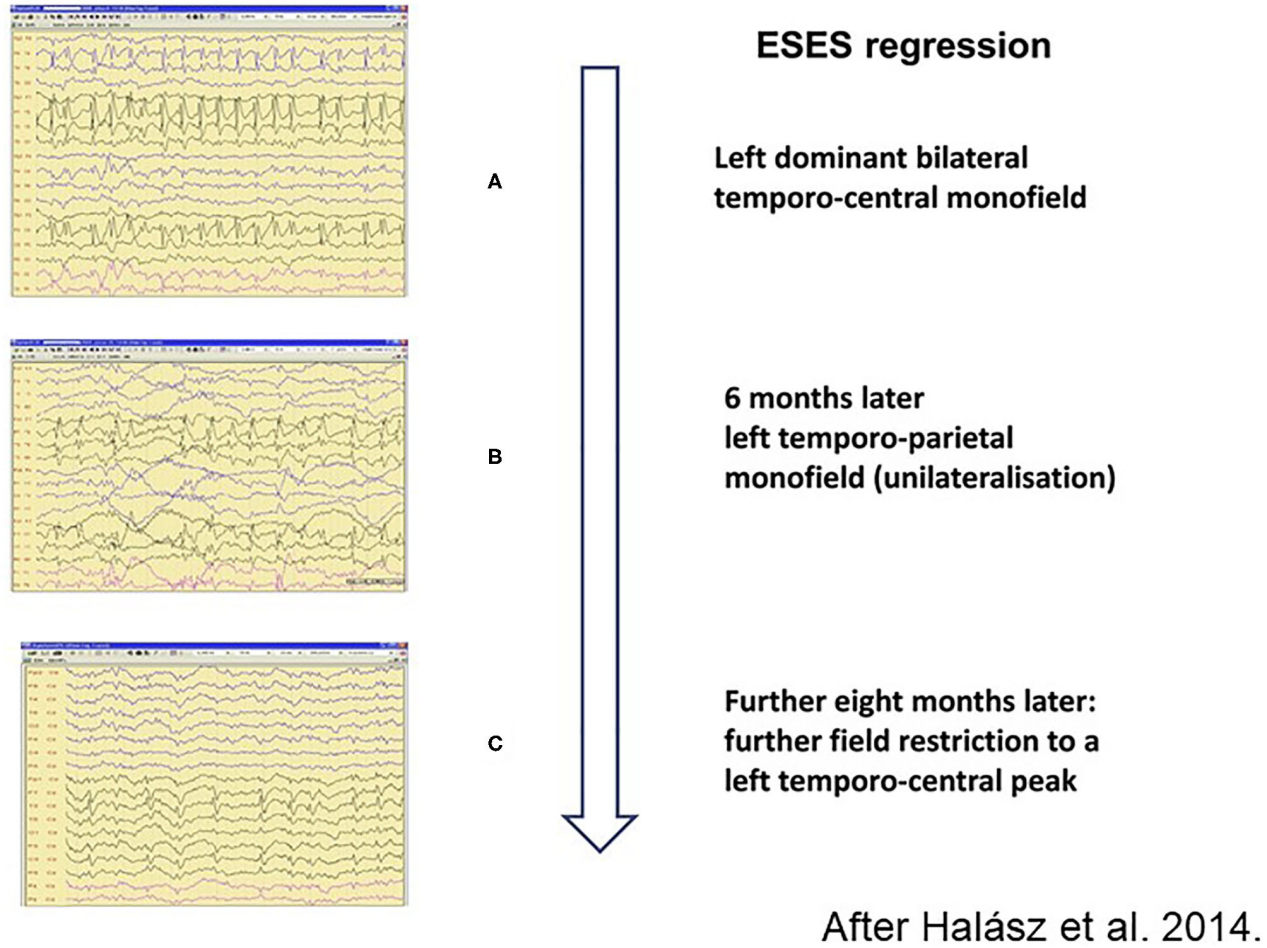
Regression of spiking during remission of electrical status epilepticus in sleep (ESES). **(A)** Left dominant bilateral temporo-central monofield. **(B)** 6 months later left temporo-parietal monofield (unilateralisation), **(C)** Further 8 months later: more field restriction of the left temporo-central peak. Perpendicular arrow represents the direction of regression process.

The spike-potentiation in NREM sleep is stronger in LKS/ESES than on the simple IFCE-end of the spectrum. The 85% coverage of NREM sleep by IED as a diagnostic criterion for ESES is not rigorously required anymore (206). A significant regional or global IED-activation is considered sufficient for making the diagnosis (207). The degree of sleep-activation changes during the evolution and revolution of ESES (146) (Figure 7) and the amount of spikes correlates with the cognitive decline.

#### Neuroimaging Features Underlying CTS

The fast development in neurodevelopmental research and neuroimaging allowed studying the abnormalities underlying the neuropsychology changes of IFCE children. Using diffusion tensor imaging, Ciumas et al. (208) showed local brain maturational deficits in the form of white matter microstructural changes. They showed decreased fractional anisotropy and increased diffusivity in the putamen and caudate nuclei on the side of the spike focus of affected children, compared with controls. They described different syndromic entities based on the structural alterations and cognitive deficit-type. Garcia-Ramos et al. (209) studied the cognitive abilities, cortical thickness and subcortical volumes of 8–15-year-old IFCE patients with CTS. Children with 2 years persistent CTS and cognitive deficits had thinner or thicker cortical regions and larger putamen of on both sides compared with healthy controls.

Shakeri et al. (210) prospectively analyzed RE children's cognitive status in line with morphology changes. Those with bilateral CTS showed significant volume-reduction in the right caudate nucleus and additional minor changes in the putamen, compared to controls.

Recently Vaudano et al. (211) studied 8,950 CTS discharges recorded in 23 patients with a mean number of 255 CTS/patient. Their results strongly support that CTSs cause malfunctioning of language networks even in wakefulness.

Another study (212) performed in an atypical RE group found overlapping features of RE and ESES/CSWS patients supporting PN conditions' spectral togetherness.

The works of the Montreal Neurological Institute revealed a far-reaching connectivity disorder behind CTS (213). Congruently, Maharathi et al. (214) confirmed that the interictal network has distinct topography compared to the seizure network.

#### Epilepsies Without Seizures

PN epilepsies display hardly any seizures. This may be due to the closing slow waves of CTS inhibiting a longer depolarization of cortical neurons necessary for the evolvement of a seizure (112). This again, makes the “true” epileptic nature of CTS dubious.

#### The Overlap With AE

Several features connect idiopathic generalized (IGE) and PN epilepsies. Thirteen to forty percent of IFCE patients exhibit bilateral spike-waves (SW), modulated by sleep and arousal, as in absence epilepsy (215–217). Degen and Degen (218) found SW even more frequently, in two third of 43 rolandic epilepsy (RE) children and in one third of their siblings. The possibility of a common genetic background of CTS/IFCE and SW/IGE has been raised by several authors (142, 219, 220).

## Summary

We collected clinical experiences and new research data on the epilepsies of the perisylvian network (PN) after our first trial to synthetize knowledge about these system epilepsies (112). The spectrum of idiopathic, age-dependent, regional, interrelated syndromes share the derailment of NREM sleep plasticity and a genetic background. The centrotemporal spike (CTS) is an endophenotype of the whole spectrum. The semiology of seizures reflects the PN system's communication-related functions. It seems convincing that the cognitive impairment and the amount of interictal epileptiform discharges (IEDs) correlate. Abundant IEDs and low seizure proneness feature each member of the spectrum.

NREM sleep-enhancement of CTS appears to mark the severity and outcome in each phenotype of the spectrum linking them in a continuum. PN conditions provide examples of epileptic cognitive impairment without seizures or brain damage, due to the IEDs that interfere with plastic sleep functions. PN epilepsies make models highlighting the pathophysiology of epileptic encephalopathies and revealing unknown pathways of cognitive impairment. They help to understand early childhood developmental epilepsies and the so-called epileptic encephalopathies.

We aimed to follow how idiopathic focal childhood epilepsies transform to epileptic encephalopathies in NREM sleep. There is a saccade of events: In the first phase with no seizures, CTS is a dubious sharp wave with no high frequency oscillation (HFO)-“crown” (185). In this phase, CTS may be a pure sleep oscillation reflecting anomalous development. Secondly, in case of epileptic transformation, CTS would convert to an epileptic sharp wave “crowned” by HFO. In the third phase, through a more important- mainly quantitative - transformation CTS may turn to more frequent and higher amplitude spikes of ESES or LKS.

The newly described constituents of PN epilepsies are ripples on the top of the CTS in the whole spectrum of IFCE. The absence, presence, prevalence and some qualitative features (e.g., amplitude) of ripples indicate the severity of the syndrome, especially the cognitive impairment.

## Post-Injury Epilepsy as the Model of Epileptic Transformation Related to Plasticity

After any brain insult, acute generalized seizures may occur within the first days post trauma, heralding the development of recurrent seizures and a later epilepsy. Although acute seizures are well manageable with antiepileptic drugs, the risk of chronic epilepsy remains.

Cerebral insults related to head-traumas, cerebrovascular accidents or other brain damages cause 20–60% of epilepsies (221). Most patients with an early acute seizure will have a second one in the next 2 years, however, 25–40% longer-term remission rate have been reported (222).

### Commonalities in Epileptogenesis Related to Different Brain Injuries

In January 2018, a group of scientists issued a review “to examine the evidence for possible commonalities in epileptogenic processes” related to different causes of human acute brain injury and acquired epilepsy. They explored the question, whether evidence from animal models was adaptable for human post-injury epilepsy and its prevention (223). We will use their statements to summarize the state-of-the-art knowledge about the genesis of posttraumatic epilepsy (PTE) and investigate, whether it is valid in other groups of epileptic disorders, too.

### The Features of Acute Brain Injury and Relations With Later Epilepsy

Spreading depression, acute non-convulsive seizures, periodic discharges or disturbances of consciousness including coma, are common symptoms in a variety of acute brain injuries. Non-convulsive seizures occur in 1/4–1/5 of cases; the incidence correlating with the size of cortical involvement, intra-parenchymal blood, convulsive seizures and coma. Acute electrographic seizure clusters, electrical status epilepticus or periodic lateralized discharges occurred more often in those patients progressing to chronic epilepsy later, regardless to the type of brain injury.

#### Common Structural Neuropathology Findings in Some Human Focal Epilepsies

In the post-injury epileptic transformation process, microglial activation and subsequent astrogliosis are common, suggesting a significant role of astrocytes in epilepsy. Heterotopic neurons named “mild malformation of cortical development” occur in the white matter. White matter angiopathy is also frequent, associating with early seizure onset and long seizure duration. The white matter changes might be traces of the hypothesized seizure network possibly involved in epileptogenesis.

#### Commonalities in the Structure and Localization of the Epileptic Networks

Stereotyped propagation patterns of epilepsy occur in some busy systems as the thalamocortical circuit and the hippocampal system. The direct injury of some cortical drivers may trigger the hippocampus in association with the limbic and thalamocortical circuits. Hippocampal injuries are extremely prone to produce chronic epilepsy.

#### Commonalities in the Reorganization After Brain Injuries

The most conspicuous reorganization pattern in status epilepticus models is cell loss, typically affecting GABAergic interneurons. Reactive synaptogenesis and axonal sprouting result in the molecular reorganization of glutamatergic and GABAergic subunits.

#### Signaling Pathway Commonalities

The four known pathways associated with epileptogenesis or progression of epilepsy after several types of cerebral insults are the followings:

- The Janus kinases/signal transducer and activator of transcription proteins (JAK/STAT) pathway regulating cell proliferation, differentiation, neurogenesis, learning and memory. It mediates the decrease of GABA A receptors in alpha1 subunits after experimental status epilepticus and traumatic brain injury, contributing to the increase of excitability and subsequent epilepsy in the hippocampus. The mammalian target of rapamycin complex1 (mechanistic target of rapamycin complex1 (mTORC1) pathway shows over-activation in the epileptogenic area associated with structural and dysplastic lesions in infantile spasm patients. The TORC1 inhibitor rapamycin had anti-seizure effect.
- An interaction of brain-derived neurotrophic factor with tropomyosin receptor kinase B (trkB) participates in fundamental cellular processes, neurotransmitter release and synaptic plasticity. Altered trkB signaling underlies epileptogenesis in kindling model.
- A trkB inhibitor targeting the Phospholipase C-γ1 (PLCy1) signaling pathway could prevent the development of epilepsy in some animals if administered during the latent period.

### The Mechanism of Epileptogenesis in Traumatic Brain Injuries Based on the Isolated Cortex Model

The Steriade School studied posttraumatic epileptogenesis in the nineties, Timofeev and co-workers continued this research. The basic fact was that isolated neo-cortical areas were hyper-excitable with local suppression-burst activity (22). Houweling et al. (224) from the same group, hypothesized, that the bursts had a homeostatic function compensating and stabilizing low neuronal activity after deafferentation. The results supported a role of homeostatic synaptic plasticity as a novel mechanism in posttraumatic epileptogenesis.

Avramescu and Timofeev (9) studied cats for 2-, 4, and 6 weeks with intra-, and extracellular electrodes after a large transection of white matter underneath the suprasylvian cortex (producing the isolated cortex model). They compared EEG-parameters with those in the presurgical state.

**Figure 8:**
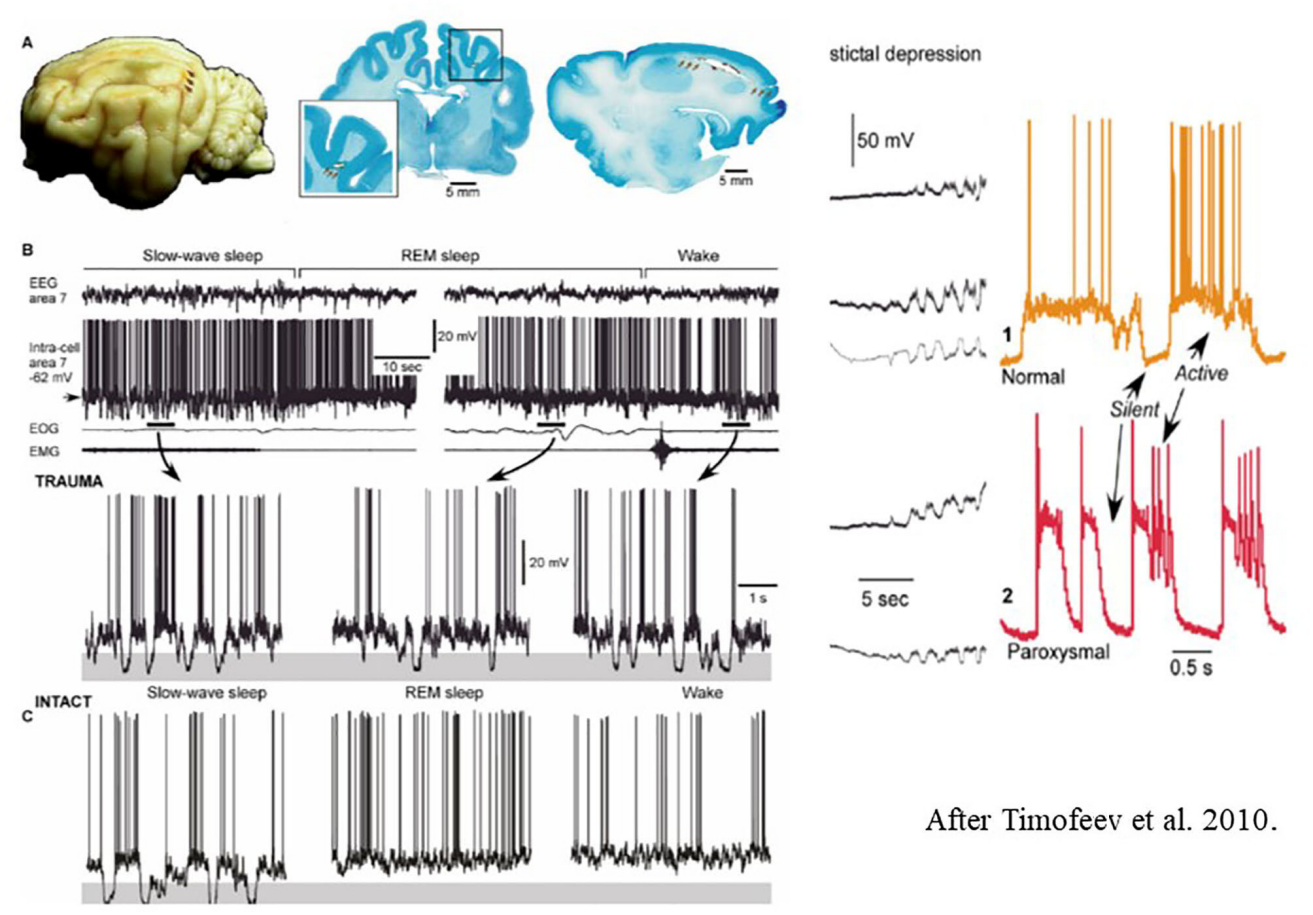
Left: **(A)** Long-lasting silent periods in all states of vigilance in the undercut cortex of cat. Localization of undercutting shown by arrows on the cat brain- **(B)** Inracellular field potential recordings during different states of vigilance. Note the presence of large amplitude hyperpolarizations (shadowed areas) [after Timofeev et al. (225)]. Right: Electrocortical seizure. Intracellular recordings. The colored shadowed areas are expanded in the right insert. During slow oscillations, the neurons oscillate between silent (hyperpolarized) and active (depolarized) states. **(C)** Intact sleep and intact sleep and wake oscillations. See that hyperpolarization present only during slow oscillations, compared with the posttraumatic state.

They revealed two parallel processes: one with weak neural activity resulting from de-afferentation, and another supposedly compensatory one with increased synaptic efficacy. This compensatory change, due to a dysfunctional homeostatic drive may increase excitability to an epileptic level and precipitate the development epilepsy (Figure 8).

In this phase, pathological plasticity with longer lasting silent periods and more suppression-burst discharges dominate. The role of silent periods in NREM sleep needs further human studies.

## Conclusions

Research data suggest a common mechanism of postinjury epileptogenesis. Several arguments support the role of homeostatic plasticity, exaggerating and derailing to an epileptic level in the injured brain. Thus, a healing process overshoots the mark and turns pathologic.

We saw that the epileptic transformation underlain by various first hits in certain big epileptic syndromes was similar. This transformation mostly affects those neuronal networks involved in plastic functions like memory or high-level human communication. The reversal of functions from restitution to epilepsy is particularly conspicuous in postinjury epileptogenesis.

## Discussion

In this paper, we tried to apply the system epilepsy concept in four types of epilepsy, to study epileptic transformation (epileptogenesis). The system-epilepsy concept had been suggested (12, 13) and applied to certain epilepsy syndromes (14) earlier. The well-known epilepsy-types we chose appear in the developmental periods of affected individuals. We considered these epilepsies suitable to study because of the high number of patients and because they affect well measurable brain functions allowing to evaluate epilepsies' impact. We overviewed the effect and outcome, taking into account the affected life-periods.

We conceived epileptogenesis as a durable process whatever the precipitator insult had been. Epileptic transformation would be an inherent distortion of brain-functions associated to plastic NREM sleep functions. In the epilepsies we reviewed, one may recognize a common way of transformation, normal sleep patterns turning epileptic. The differences of affected systems and their relation to sleep determine the way and type of transformation, the resulting epilepsies reflecting the features of the given system. We considered postinjury epilepsy a good model of epileptic reorganization although its sleep relations have been less studied.

The epileptic transformation in the twin-systems regulating vigilance (shift to sleep and arousal from sleep) is peculiar. It provides insight into the mechanism of those reflex epilepsies, in that the normal functioning of an epilepsy-prone system (without a clear stimulus) may trigger seizures.

The interesting link between arousal parasomnias and nocturnal frontal lobe epilepsy is worth discussing. The first one is a sleep disorder; the second one is epilepsy, but they show similar sleep-relatedness, seizures/episodes with analogous symptoms, and they share a common genetic background. Both conditions seem to have a microarousal-related sleep-dissociation mechanism, too, a high amount of autonomic and motor arousals co-occurring with local sleep in the dorso-frontal cognitive network. It is tempting to propose an epileptic transition from APs to NFLE, but a convincing proof is missing yet.

In those epilepsies we reviewed, interictal discharges were more important in the sleep-associated transformations than seizures. In recent literature, we witness many works emphasizing the important role of IEDs in the association of NREM sleep and epilepsy. IEDs are obviously present in all epilepsies, but their role is uncertain and their relationship with seizures is unclear. The discharge rate of IEDs is not a measure of seizure threshold while the old dogma that epilepsy does not exist without seizures is not valid anymore. Recently, with the development of fMRI, we start to understand the hitherto hidden widespread network and effect of IEDs, including centrotemporal spikes in subcortical structures (208–210, 214, 226, 227). Experimental data show that in the process of epiletogenesis, IEDs appear first and seizures come later (228).

A big mass of evidence on IEDs' influence on the cognitive sphere came from the recognition of idiopathic focal childhood epilepsy children's cognitive impairments (143), which is even more severe in the malignant encephalopathic variants ESES/LKS of the spectrum. Recently, it has been demonstrated (185, 186) that CTS may undergo an epileptic transformation i.e., associating to HFO, and further to NREM sleep-related continuous spiking (229). Albeit IEDs interfere with sleep plastic functions, the actual cognitive loss origins from the loss of NREM sleep-related synaptic plasticity.

The place of IEDs in epileptogenesis as well as their impact on brain functions have remained challenging issues. CTS likely signalize time-limited regional retardation of cortical development and increased cortical excitability. Coupling with ripples, CTS can progress to epilepsy. Without this epileptic evolution, normal development may restore, while an epileptic evolution turns CTS to a pathological agent interfering with cognitive development. IEDs may be the functional building stones of epilepsies and mark the stage of the epileptic process both in progression and in regression.

It is unclear whether the paramount role of NREM sleep seen in major childhood epilepsies, applies to those epilepsies starting in adulthood in periods of less intense plastic functions, e.g., the large group of postinjury epilepsies. These conditions make excellent models for a common process of epileptogenesis in general; they just differ in that the injury determines its site within the brain.

We discussed the role of NREM sleep in epilepsy from the aspects of sleep-related synaptic homeostasis and plasticity. Homeostatic plasticity is a compensatory (healing) process, correcting or recovering cell loss and functional decrease that may go together with the increase of excitability. The increase of excitability may reach a paroxysmal (epileptic) level. In our system epilepsies, we found supporting examples of this attractive theoretical model. This is a quality change where normal functioning transforms to an epileptic working mode. We propose that this process may link different epilepsies in a more or less homogenous group and connect them with NREM sleep plastic functions.

NREM sleep potentiates epileptic phenomena, which in turn, interfere with sleep functions. This vicious circle works every night, transforming brain functions, especially during the flexible periods of development and in brain systems involved in plasticity.

## Author Contributions

PH had the idea, then both authors participated in making the concept, the main messages, and in the formulation of the paper.

## Conflict of Interest

The authors declare that the research was conducted in the absence of any commercial or financial relationships that could be construed as a potential conflict of interest.

## Footnotes

^1^However catchy, this metaphor rests on the idea, that epilepsy is a foreign outside force raping the brain. The assumption that epilepsy would be a derailment of inherent plasticity seems more plausible.

^2^Gastaut type occipital epilepsy (111) is the smallest group (2–7%) of IFCE. Its IEDs and ictal symptoms unanimously delineate an occipital localization. It is an epilepsy of later childhood (the age at onset is 3–15 years with a peak at 8–11 years) compared to RE and PS. The IEDs are less variable than in RE and PS. There is some overlap in the IEDs and age dependency with RE and PS, but the transformation to ESES/LKS is less documented. Therefore, Gastaut type idiopathic occipital childhood epilepsy seems not to be part of the PN spectrum, and we will not incorporate it in this overview (112).

^3^In several epilepsies, spikes couple with the upstate of sleep slow waves, while CTS –the spikes of PN epilepsies-, couple with sleep spindles. The reason of this dichotomy y - coupling of IEDs with spindles or slow waves, is unknown. Regarding its high-level functions, it seems likely that PN is a human neo-formation; which would be reflected by phylogenetically new epilepsy-types as well. These epilepsies link to human communication functions, strongly coupling with sleep spindling rather than to slow waves.

^4^There is a confusion in the terminology of the encephalopathic variants of IFCE, especially regarding the terms ESES and continuous spike-waves in sleep (CSWS) as well as the relation of LKS and ESES (145). One cause of the confusion may be the interpretation of the EEG changes in ESES. Several authors consider it a synchronous bilateral spike-wave pattern, but abundant data support the uni-hemispheric, focal/regional localization of discharges (112, 146).

## Abbreviations

Ach: Acetylcholin
ADNFLE: autosomal dominant nocturnal frontal lobe epilepsy
AE: absence epilepsy
AP: arousal parasomnia, NREM parasomnia (e.g., night terror, sleep walking)
CA: Cornu ammonis
CAP: cyclic alternating pattern
Corticothalamic neuron: TC neuron
CTS: centrotemporal spike
CSWS: continuous spike-waves in sleep
EEG: electroencephalography
ESES: Electrical status epilepticus in sleep
fMRI: functional magnetic resonance imaging
GABA: gamma-aminobutyric acid
HFO: high frequency oscillations including “slower” (100–200 Hz), and higher (above 200 Hz) frequency ranges. Synonym, ripple; slow and fast, too
HS: hippocampal sclerosis
IED: interictal epileptiform discharge
IFCE: Idiopathic focal childhood epilepsy
IGE: Idiopathic generalized epilepsy
JME: juvenile myoclonic epilepsy
LKS: Landau-Kleffner syndrome
AEA: acquired epileptic aphasia
MTLE: medial temporal lobe epilepsy
nAchR: nicotinic acetylcholine receptor
NFLE: nocturnal frontal lobe epilepsy
nRE: Thalamic reticular nucleus
NREM sleep: non rapid eye movement sleep
PN: Perisylvian human communication network, Perisylvian network
PS: Panaiyotopoulos syndrome
RE: Rolandic epilepsy, centrotemporal epilepsy (earlier term: benign centrotemporal epilepsy)
REM sleep: rapid eye movement sleep
SHE: sleep related hypermotor epilepsy
SPW-R: Sharp wave ripple
SW: Bilateral synchronous spike and wave discharges.
